# Linking Animal Feed Formulation to Milk Quantity, Quality, and Animal Health Through Data-Driven Decision-Making

**DOI:** 10.3390/ani15020162

**Published:** 2025-01-10

**Authors:** Oreofeoluwa A. Akintan, Kifle G. Gebremedhin, Daniel Dooyum Uyeh

**Affiliations:** 1 Department of Biosystems and Agricultural Engineering, Michigan State University, East Lansing, MI 48824, USA; akintano@msu.edu; 2 Department of Biological and Environmental Engineering, Cornell University, Ithaca, NY 14853, USA

**Keywords:** animal feed formulation, decision support systems, milk quality, milk yield, precision nutrition

## Abstract

The global demand for high-quality animal products, particularly dairy, has intensified the need for more precise and efficient livestock feed formulation. This review connects data-driven decision-making in optimizing feed formulation to enhance milk quantity and quality while addressing animal health implications. Modern feed formulation has evolved into a sophisticated, data-centric process by integrating diverse data sources such as nutritional databases, environmental data, and animal performance metrics. Leveraging advanced analytical techniques, such as machine learning and optimization algorithms, have created highly accurate feed formulations tailored to specific livestock needs. These innovations increase milk yield and contribute to developing dairy products with higher nutritional value. Decision Support Systems play a complementary role by offering real-time decision-making capabilities, enabling farmers to make data-informed adjustments composition based on changing conditions. However, despite its potential, the widespread adoption of data-driven feed formulation faces challenges such as data quality, technological limitations, and industry resistance, mostly disjointed processes. The objectives of this review are: (i) to explore the current advancements and challenges of data-driven decision-making in feed formulation, focusing on its connection to milk quantity and quality, and (ii) to highlight how this optimized feed formulation strategy improves sustainable dairy production.

## Simple Summary

Precision feed formulation is essential to improve livestock health, productivity, and milk quality, which directly affects animal health. The feed can be tailored to better meet livestock needs and enhance their growth and production by utilizing data-driven techniques such as machine learning and advanced decision-making systems. These advancements help improve milk quality and promote sustainability in dairy farming, though challenges such as data quality and industry adoption remain. These innovations contribute to better animal health and more efficient dairy production.

## 1. Introduction

The growing global demand for high-quality animal products, especially dairy, underscores the need for precision livestock feed formulation to enhance livestock health, productivity, and milk quality. The nutritional composition of animal feed directly affects the physiological well-being of livestock, which is essential for optimal growth and reproduction [1]. A well-balanced diet ensures that livestock receives adequate protein, energy, vitamins, and minerals, vital for maintaining animal health and productivity [2]. Research indicates that the quality of feed significantly influences the quality of milk produced, as the diet’s nutritional profile directly correlates with the composition of milk, including its fatty acid profile and overall nutrient content [3,4]. Feed formulation also affects livestock’s health by minimizing the risk of diseases and improving their immune response [5]. For example, precision feeding techniques that tailor diets to meet the specific nutritional needs of animals can reduce feed wastage and enhance nutrient absorption, thereby promoting better health outcomes [4,6]. Furthermore, high-quality feed ingredients can improve milk quality, characterized by higher concentrations of beneficial nutrients such as omega-3 fatty acids and conjugated linoleic acid (CLA), linked to various human health benefits [4,6]. Conversely, poor feed quality or imbalanced rations can lead to health issues in livestock, such as metabolic disorders, which can ultimately affect milk production and quality [7,8].

Traditional feed formulation methods have long been the backbone of livestock nutrition yet often fail to meet modern agricultural and nutritional demands. These conventional approaches typically rely on fixed ratios and generalized nutrient profiles that do not account for the variability in ingredient quality, animal requirements, and environmental conditions. As a result, traditional methods can lead to inefficiencies in nutrient utilization, suboptimal animal performance, and increased feed costs [9]. Moreover, the reliance on manual calculations and simplistic linear programming techniques limits the ability to adapt to the dynamic needs of livestock, particularly in the face of changing market conditions and evolving consumer preferences for higher-quality animal products [10]. In contrast, the rise of data-driven methods has transformed feed formulation by leveraging advanced computational techniques and real-time data analytics to optimize feed for maximum efficiency and productivity. These modern approaches employ nonlinear programming and machine learning algorithms to develop more precise and adaptable feed formulations, effectively addressing the specific nutritional requirements of livestock [11]. For instance, nonlinear programming allows for considering diminishing returns in nutrient absorption, which is often overlooked in traditional linear models [10]. This results in formulations that not only enhance growth rates and milk production but also improve the overall nutrient profiles of the feed, thereby benefiting both animal health and product quality [1]. Furthermore, data-driven methods facilitate the incorporation of diverse feed ingredients, including local and alternative sources, which can enhance the nutritional value of feed while reducing costs [12]. Analyzing the digestibility and bioavailability of various ingredients enables the formulation of diets that maximize nutrient retention and minimize waste [13]. Integrating functional feed additives, such as probiotics and prebiotics, into formulations also reflects a shift towards more holistic approaches that prioritize animal health and sustainability [14]. The transition from traditional to data-driven feed formulation is not merely a technological upgrade; it represents a fundamental change in how the livestock industry approaches nutrition. While traditional feed formulation methods have served their purpose, they are increasingly inadequate in addressing the complexities of modern livestock nutrition. The emergence of data-driven techniques offers a promising pathway to optimize feed formulations, enhance livestock productivity, and improve the nutritional quality of animal products, benefiting both producers and consumers.

This review highlights the importance of data-driven decision-making techniques in advancing feed formulation to enhance milk quality and quantity. It also explores the role of identifying optimal feed ingredients and environmental conditions, their impact on nutritional composition, and their broader implications for animal health. Additionally, it addresses the challenges and prospects of implementing data-driven strategies in feed formulation.

### Materials and Methods

For a comprehensive review of data-driven feed formulation approaches to improve decision-making for enhancing milk yield, quality, and dairy animal health, we utilized the NLP tool Scite.ai in our literature search [15]. Scite.ai is a bibliometric platform that aids in conducting literature reviews by offering additional information on citations. It compiles citation data from diverse sources, including academic publishers, peer-reviewed journals, and open-access repositories. These data are collected through indexing extensive collections of research articles such as PubMed, CrossRef, and platforms like arXiv. Scite.ai classifies citations into three categories: supporting, contrasting, or mentioning. This categorization provides contextual information on how a specific work is referenced, highlighting its validation, critique, or acknowledgment. By delivering citation insights, Scite.ai aids the understanding of a publication’s scholarly impact and relevance.

**Figure 1:**
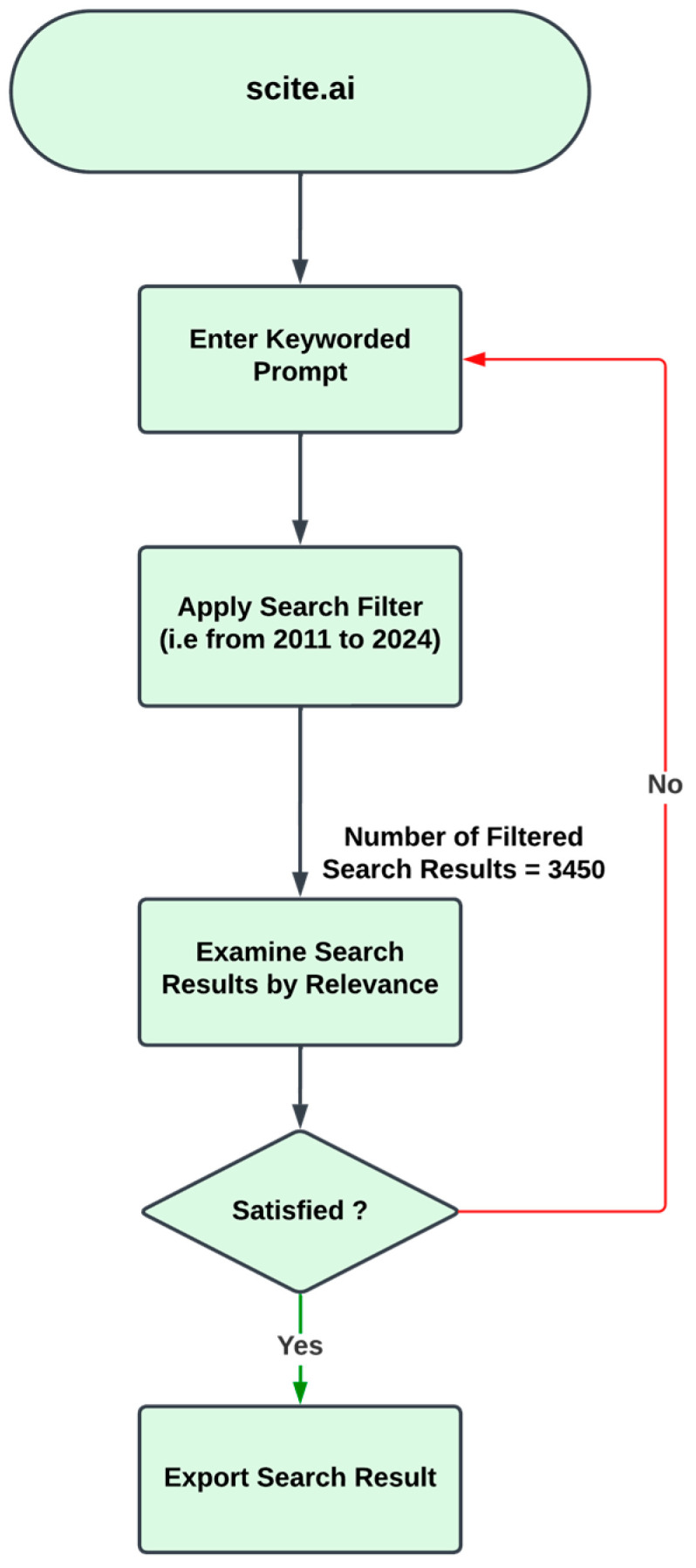
Procedure for literature extraction and review with an NLP tool.

**Figure 2:**
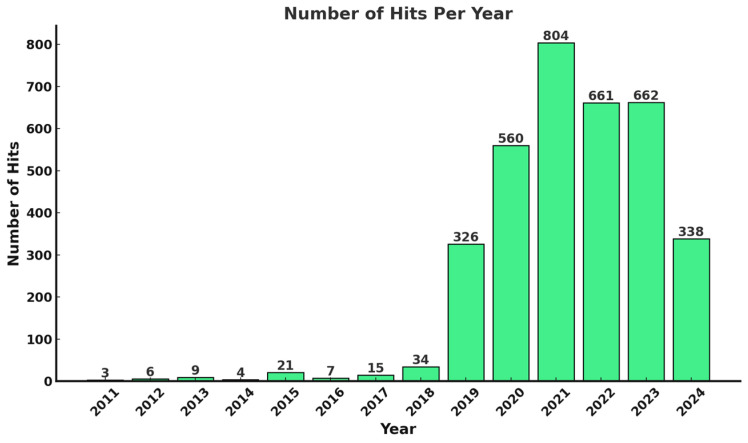
Paper hits per year based on scite.ai search results for the keywords “Animal Feed Formulation + Milk Yield”.

Figure 1 illustrates the decision-making process and outcomes for generating search results from a keyword-based prompt, specifically using the keyword “Animal Feed Formulation + Milk Yield”. Filters such as year range and specific journals were applied to reduce the volume of the results and refine the focus of the search. Figure 2 displays the number of hits per year based on the specified keywords. The filtered results were then evaluated for their relevance to the focus topic. If deemed satisfactory, the results were exported as a CSV file for further screening to determine which literature should be included. Filtered search results were only accepted if they aligned with the topic; otherwise, keywords were adjusted to reduce the results volume and generate more relevant outcomes. This iterative process was applied to multiple keywords, including combinations like “data-driven decision-making”, “feed formulation”, “milk quality”, “precision agriculture”, “decision support systems”, “milk yield”, “animal nutrition”, and “environmental conditions” to locate relevant studies.

The final selection of papers for the review was based on their relevance to identifying feed ingredients and environmental conditions that enhance milk yield and nutritional composition, animal feed formulation targeted at improving milk production quality, data analysis techniques for optimizing feed formulations, and data-driven decision-making methods adapted for feed formulation. Papers were screened using their titles, abstracts, and full texts, when necessary, to ensure a thorough evaluation of their relevance to the study’s objectives.

## 2. Foundation for Data-Driven Feed Formulation

Data-driven feed formulation has become increasingly important in the dairy industry, allowing for more precise nutrition tailored to the specific needs of livestock. This precision not only enhances milk production but also improves the nutritional quality of the milk produced. Farmers can optimize feed formulations using various data sources based on available information about animal health, environmental conditions, and nutritional requirements. The data collected from these diverse sources forms the backbone of data-driven feed formulation. By analyzing nutritional compositions, feed intake patterns, performance metrics, digestibility, economic factors, and genetic influences, researchers and practitioners can develop optimized feeding strategies that significantly enhance milk yield in dairy cattle. Several case studies illustrate the effectiveness of data-driven approaches in improving milk yield and nutrient quality. For instance, Bortacki et al. [16] found that increasing milking frequency significantly improved milk yield in high-producing dairy herds, emphasizing the importance of data on milking schedules and their impact on production [2]. Similarly, Sharma et al. [17] utilized decision tree analysis to predict 305-day milk yield in crossbred cattle, demonstrating how data analytics can identify key factors influencing milk production [3]. Additionally, Parmer et al. [18] highlighted the role of climatic factors on milk production, showing that data on environmental conditions could inform feed formulation strategies to optimize yield during different seasons. The case studies presented illustrate the practical applications of these methodologies, highlighting their potential to transform the dairy industry. The diverse methods employed in research to optimize dairy production are detailed in Table 1, highlighting the wide range of data, metrics, and methodologies used to enhance milk yield across various cattle breeds. The table demonstrates how targeted interventions can significantly improve milk yield by integrating breeding traits, feed efficiency, and nutritional strategies.

The formulation of livestock feed has evolved with the advent of technology and data analytics. Various data sources play a crucial role in developing effective feed formulations that optimize animal health, productivity, and the quality of animal products. Nutritional databases serve as foundational resources for feed formulation, providing comprehensive information on the nutrient composition of various feed ingredients. These databases compile data from multiple studies, allowing nutritionists to access reliable information regarding the digestibility, energy content, and nutrient profiles of different feedstuffs [19]. For instance, the National Research Council (NRC) [20] and the Feed Composition Tables are widely used references offering standardized nutrient values for various ingredients. Utilizing these databases enables formulators to produce balanced rations that meet the specific dietary requirements of livestock [19]. Wilkinson and Garnsworthy [21] highlight that diets based on grazed grass and silage resulted in higher human-edible feed efficiency than those with high concentrate levels. This indicates that the choice of feed ingredients, informed by nutritional data, directly influences animal productivity and milk production’s environmental impact. Integrating sensor technology into livestock management has improved how data are collected and utilized in feed formulation. Sensors can monitor various parameters, including feed intake, rumen pH, and animal behavior, providing real-time insights into the nutritional status and health of livestock. For example, electronic feed bunk sensors can track individual animal feed consumption, allowing for precise adjustments to be made in feed formulations based on actual intake patterns [22].

**Table 1:** Methods for Enhancing Milk Yield Across Dairy Animals.

Methods		Data/Metrics Used for Milk Yield Optimization	Key Findings	Source
	Regression analysis, Multivariate analysis, Genomic analysis	Feed intake, Milk yield, body weight, and energy efficiency.	Feed efficiency was highly correlated with milk yield. Cows with higher milk production per unit of feed intake were more efficient.	[23]
	t-tests to compare productive performance parameters	Pure and Graded Siri cattle milk yield, lactation length, dry periods	Significant differences in lactation performance, with graded cows showing higher milk yields.	[24]
	Two-way ANOVA	Milk Yield, Milk Composition, Feed Intake	The use of these strains promoted a significant increase in milk production and overall production efficiency.	[25]
	Linear mixed effects model	Milk Yield and Supplemented Feed Intake	Cows showed improved milk yield with specific dietary supplements.	[26,27]
	SAS MIXED procedure	Temperature Humidity Index (THI), Feed Intake, Digestive efficiency, Milk yield and compositions	Complete confinement enhanced milk production and feed efficiency.	[28]
	PROC MIXED analysis of milking schedules	Milk yield, composition, dry matter intake, energy balance, and reproductive metrics.	Cows on the high-energy diet had a higher milk yield than those on the standard diet.	[29]
	Decision tree analysis	Age, lactation length, calving season, and milk yield	The lactation length was the most significant factor affecting milk yield, followed by parity, age, and calving season.	[18]
	Partial Least Squares-Discriminant analysis	Dairy Herd Improvement data (Milk yield and milk composition)	A higher average milk yield compared to Pure Holstein.	[30]
	One-way ANOVA and Principal component analysis	Pasture intake, grazing behavior, feed quality	Daily milk yield was highest in cows fed with soya hulls and beet pulp, while protein and fat content varied across groups.	[31]
	One-way ANOVA	Physiological parameters, body characteristics, lactation stage, Milk composition, yield, and Temperature.	Milk yield decreased significantly under high THI conditions, with a drop of 14% when THI moved from low to high levels.	[32]
	GLM procedure of SAS and Duncan test	Feed Intake, Milk Yield, and milk production efficiency (MPE)	Supplements improved milk production efficiency, with higher MPE values indicating better nutrient absorption from dry matter intake. However, the feed supplements had no significant effect on milk quality.	[33]
	Two-way ANOVA Model, Tukey’s Test	Milk Yield and Milk Composition	Milk yield was highest in the sixth parity hybrids during the summer.	[34]
	One-way analysis of variance (ANOVA)	Body weight, Nutrients intake, Milk yield, Milk composition, Feed conversion efficiency, and Cost of feeding	Supplementation with fibrolytic enzymes improved feed efficiency and milk yield in early lactation cows.	[35]
	General linear model (GLM) procedure	Milk yield, Feed Intake	Goats fed with Asperozym-supplemented rations had the highest average daily fat-corrected milk yield.	[36]
	PROC MIXED procedure	Milk yield and milk composition data (fat, protein, dry matter, lactose, and somatic cell count)	Factors like farm management practices, birth season, and lactation stage notably influenced milk yield and composition variability.	[37]
	Post-hoc analysis	Milk yield, milk composition, feed intake, and digestibility	Goats fed with the formaldehyde-treated sesame meal diet had the highest milk yield	[38]
	Mixed linear model -PROC GLIMMIX	Milk yield, milk composition (e.g., fat, protein, total solids), and mineral profiles	The inclusion of 40% artichoke by-product silage in the diet did not negatively affect milk yield.	[39]
	Nested ANOVA, Bayes factor analysis	Milk yield, Feed Supplementation Intake	The antioxidant-supplemented group showed a significant improvement in average daily milk yield.	[40]
	Stratified Sampling	Strata weights, Milk yield	Diseases like milk fever significantly reduce daily milk yield.	[41]
	Real-coded hybrid genetic algorithm (RGA) combined with Linear Programming (LP)	Body Weight, Feed Cost, Milk Yield, Fat percentage, Feed intake, and Nutrient requirement values	RGA can effectively optimize feed formulations to minimize costs while maintaining milk yield.	[42]
	Unpaired t-tests	milk yield, calf birth weight	The mother’s nutritional intake primarily influenced milk yield and early calf growth during pregnancy.	[43]
	Stepwise Regression analysis	Climatic data, Daily Milk Yield	Milk production peaks in cooler months, indicating seasonal feeding adjustments.	[19]
	ANOVA, Regression analysis, F-tests	Milk yield, body weight, and hump weight	Fermentable carbohydrates in excess compromised milk yield while promoting weight gain and hump size.	[44]
		**Dairy Animal Species**		
		Cows		
		Crossbred Cattle		
		Goats		
		Buffaloes		
		Reindeer		
		Triple-Bred Cattle		
		She-camel		

This data-driven approach facilitates the identification of underperforming animals and enables targeted nutritional interventions, leading to improved productivity and efficiency [45]. Historical animal performance data are another critical resource for feed formulation. By analyzing past performance records, nutritionists can identify trends and correlations between feed formulations and animal outcomes, such as growth rates, milk yield, and reproductive performance. These data can be used to refine feed formulations over time, ensuring that they remain effective in meeting the evolving needs of livestock. For instance, a study by St-Pierre and Weiss [46] demonstrated how historical performance data can inform the development of customized feeding strategies that optimize milk production in dairy cows. Such analyses contribute to improving feed formulations and overall herd management practices. Environmental factors, including temperature, humidity, and housing conditions, influence the nutritional requirements of livestock. Understanding these factors is essential for formulating diets for animal metabolism and feed efficiency variations. For example, heat stress can reduce feed intake and alter nutrient utilization, necessitating adjustments in feed formulations to maintain optimal performance [47]. By incorporating environmental data into feed formulation strategies, nutritionists can develop more resilient diets that support animal health and productivity under varying conditions [22]. For instance, real-time monitoring of rumen fermentation parameters can guide the formulation of diets that optimize nutrient absorption and minimize waste [48]. The ability to adapt feed formulations in real-time enhances overall efficiency and productivity in livestock operations.

### 2.1. Nutritional Data Collection for Data-Driven Feed Formulation

**Figure 3:**
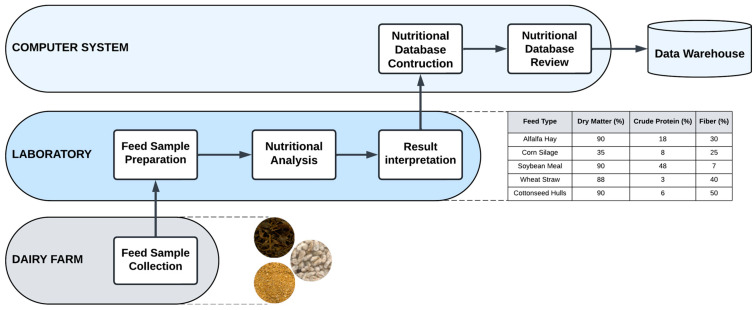
Workflow for Nutritional Data Collection.

In the formulation of animal feed, particularly for dairy cows, the collection of relevant data are paramount for optimizing nutritional strategies that enhance milk production and overall herd health. Essential data sources include feed composition tables, which provide critical information on the nutrient content of various feed ingredients, such as energy, protein, fiber, vitamins, and minerals. Accurate feed composition data are crucial for formulating balanced diets that meet the specific nutritional requirements of dairy cows at distinct stages of lactation [49]. For instance, the nutritional value of feedstuffs can vary based on their source and processing methods, necessitating the use of updated and region-specific feed composition tables [50]. Figure 3 illustrates the process of creating nutritional databases for dairy cattle. The process begins with the collection of feed samples, which are then subjected to nutritional analysis to determine their composition. This analysis includes evaluating macronutrients (proteins, carbohydrates, fats), micronutrients (vitamins and minerals), and fiber content. Following this, the nutritional requirements of dairy animals are assessed based on their physiological status (e.g., lactating, dry, or growing) and production goals, which are critical for formulating balanced diets [51,52]. Once the nutritional content of the feed and the requirements of the animals are established, the next step involves formulating the diet. This can be achieved by using linear programming models that optimize the allocation of nutritional resources to meet the animals’ needs while considering feed availability and cost [52]. The formulated diets are then implemented on the farm, where animal performance and health monitoring are essential to meet nutritional goals. Adjustments to the diet may be necessary based on ongoing assessments of milk production, feed intake, and overall animal health [6,53].

### 2.2. A Framework for Data-Driven Decision-Making in Feed Formulations

Data-driven decision-making in feed formulation is a systematic approach that leverages insights derived from data to inform and optimize decisions regarding livestock nutrition. This framework is particularly relevant in the context of dairy farming, where the integration of data analytics can enhance productivity and sustainability. A proposed framework for data-driven decision-making, initially designed for educational institutions, can be adapted for dairy farms, facilitating the application of data insights to improve feed formulations [54,55]. The data in this context progresses through four primary stages, creating a continuum from data collection to informed decision-making, following the DIKW (Data-Information-Knowledge-Wisdom) framework [56]. The first stage of this data continuum is data collection, which gathers raw facts and figures related to nutritional, environmental, and physiological parameters affecting milk production. For instance, nutritional data may include the composition of feed ingredients, while environmental data could encompass factors such as temperature and humidity, which influence feed intake and digestion [57]. Physiological data might involve metrics such as milk yield and health indicators of dairy cattle. The subsequent stage consists of transforming this raw data into information through contextualization and analysis.

Once the data have been contextualized into information, the next phase is the generation of knowledge. Knowledge represents a synthesis of useful information that dairy farmers can utilize to set specific formulation objectives, such as enhancing milk production and improving nutritional composition [58]. At this stage, farmers can employ decision support systems (DSS) that integrate algorithms and machine learning models to optimize feed formulations based on the identified objectives. The decision-making process involves evaluating trade-offs between cost, nutrition, and other relevant factors, ensuring that the chosen feed formulation aligns with both economic and production goals [59]. For example, least-cost programming (LCP) exemplifies how contextualized nutritional data can be utilized by comparing the nutrient profiles of various feed types against the specific dietary requirements of dairy cows. By integrating LCP into DSS, farmers can identify optimal feed combinations that meet nutritional needs while minimizing costs. Alqaisi and Schlecht [59] demonstrated how LCP integrates nutrient profiles and feed availability to formulate cost-effective rations tailored to milk production scenarios.

**Figure 4:**
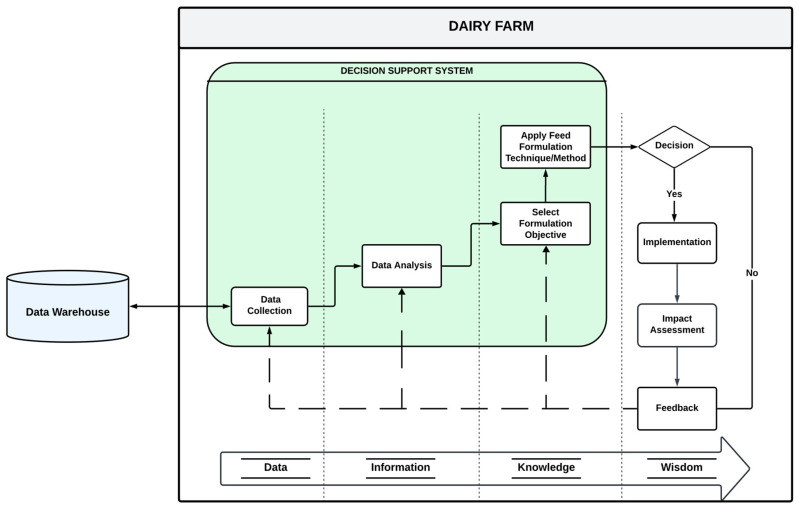
Framework for data-driven decision making in a dairy farm.

Applying knowledge in this manner signifies the effective use of data-driven insights in practical decision-making, termed wisdom for this framework. Decision support tools and systems are pivotal in facilitating this data-driven decision-making process. These tools encompass both software and hardware components that aid in data collection, processing, and analysis, thereby streamlining the decision-making workflow [55]. As illustrated in Figure 4, the evolution of data within the DSS progresses from initial collection to final decision-making, where formulation outcomes can either be accepted or rejected. Accepted formulations are implemented, and their impacts are assessed, while rejected formulations prompt feedback to the DSS for exploring alternative approaches [60]. This iterative feedback loop is essential for continuous improvement in feed formulation strategies. In conclusion, the framework for data-driven decision-making in feed formulations for dairy farms underscores the importance of a systematic approach that transitions from data collection to informed decision-making. By leveraging decision support systems and contextualizing data into actionable knowledge, dairy farmers can optimize their feed formulations, enhancing productivity and sustainability in dairy production.

## 3. Data-Driven Analytical Techniques and Tools in Feed Formulation

### 3.1. Machine Learning and Predictive Analytics in Feed Optimization

Applying machine learning (ML) and predictive analytics in feed optimization has emerged as a transformative approach in the dairy industry, enabling the generation of actionable insights that enhance feed formulation, predict nutrient requirements, and improve milk yield and quality. Machine learning models facilitate the development of optimized feed formulations by analyzing datasets that include nutritional content, animal performance, and environmental conditions. For instance, Cockburn [61] highlights that machine learning algorithms have improved the prediction of resource consumption on dairy farms, demonstrating their capability to process and analyze large volumes of data more effectively than traditional methods. Moreover, machine learning techniques can identify patterns and correlations within historical performance data, allowing for the prediction of future nutrient requirements based on past trends. This predictive capability is crucial for formulating diets that meet current needs and anticipate future changes in animal health and productivity [62]. Using recurrent neural networks (RNNs) has been shown to effectively predict calving dates and optimize feeding strategies, ensuring that animals receive the appropriate nutrition at critical stages of production [62]. The ability to predict nutrient requirements accurately is a significant advantage of machine learning applications in feed optimization. By analyzing numerous factors, such as age, weight, lactation stage, and health status, machine learning models can provide precise recommendations for nutrient intake. This predictive analytics approach helps minimize feed waste and ensure that livestock receive the optimal balance of nutrients necessary for their growth and milk production. Research by Nguyen et al. [63] demonstrates the effectiveness of combining animal and diet parameters to forecast milk production using machine learning models. Their study compared the performance of linear models with machine learning approaches, revealing that the latter outperformed traditional methods in predicting milk yield. This finding underscores the potential of machine learning to enhance the accuracy of nutrient requirement predictions, leading to more efficient feed formulations.

Integrating machine learning in feed optimization enhances nutrient prediction and directly impacts milk yield and quality. Advanced algorithms are utilized to analyze factors affecting milk production. For instance, Becker et al. [64,65] employed machine learning techniques to predict heat stress in dairy cattle, adversely affecting milk yield. By proactively managing feeding strategies based on predicted heat stress events, farmers can mitigate its impact on production. Machine learning models have also been used to analyze milk composition data, providing insights into how different feed formulations influence milk quality. Ebrahimie et al. [66] conducted a study highlighting the predictive power of milk composition features, such as lactose concentration, in diagnosing subclinical mastitis. This research illustrates how machine learning can enhance milk quality by identifying optimal feeding strategies that promote healthy udder function. Integrating machine learning and predictive analytics into feed optimization represents a significant advancement in dairy management. These technologies are reshaping the framework of dairy farming by generating actionable insights for optimizing feed formulation, predicting nutrient requirements, and improving milk yield and quality.

### 3.2. Optimization Algorithms for Cost-Effective and Nutritious Feed

The optimization of feed formulations is critical for enhancing the productivity and profitability of dairy operations. Various optimization techniques, including linear programming (LP), genetic algorithms (GA), and mixed-integer programming, have been developed to formulate feed that balances cost, milk production, and quality. It involves creating mathematical models that define the relationships between feed ingredients, nutrient requirements, and associated costs. The primary objective of LP is to minimize feed costs while meeting the nutritional needs of livestock. Bellingeri et al. [52] developed a linear programming model for the optimal allocation of nutritional resources in a dairy herd, demonstrating how mathematical computations can streamline decision-making processes in feed formulation. Their findings indicate that LP can effectively balance feed costs with nutritional adequacy, enhancing milk production and quality. Patil [67] highlighted the advantages of LP in the least-cost feed formulation for lactating cattle. The study emphasizes that while traditional feed formulation methods often overlook variability in nutrient values, LP provides a structured approach to ensure that nutrient requirements are satisfied at the lowest possible cost. This capability is particularly beneficial in environments where feed resources are limited or fluctuating, allowing dairy producers to maintain profitability while ensuring animal health. Genetic algorithms (GAs) represent another innovative approach to feed optimization. These algorithms mimic the process of natural selection to explore a wide range of potential feed formulations and identify the most effective combinations of ingredients. By evaluating multiple variables simultaneously, GAs can optimize feed formulations based on various criteria, including cost, nutrient composition, and production outcomes. Research by Zhao et al. [68] illustrates the application of GAs in optimizing total mixed ration (TMR) diets for dairy cows. The study emphasizes the importance of identifying nutritionally active substances or nutrifies within feed formulations. By employing GAs, the researchers could analyze the relationships between different feed components and their effects on dairy cow health and productivity. This approach enhances feed efficiency and contributes to improved milk quality.

Mixed-integer programming (MIP) is another optimization technique that has gained traction in feed formulation. MIP allows for the inclusion of continuous and discrete variables in the optimization process, making it particularly useful for complex feed formulations requiring specific ingredient combinations. Metwally [69] applied MIP to optimize broiler chicken feeds, demonstrating its effectiveness in balancing cost and nutritional requirements while accommodating various constraints. In the context of dairy production, MIP can be utilized to develop feed formulations that consider multiple factors, such as ingredient availability, nutritional content, and production goals. This flexibility allows dairy producers to formulate customized diets that optimize milk yield and quality while minimizing production costs. Integrating optimization algorithms in feed formulation also addresses economic factors influencing dairy operations. As highlighted by Duguma and Janssens [70], the low availability and quality of feeds can significantly impact the productive and reproductive performance of dairy cows. Dairy producers can make informed decisions that enhance feed efficiency and reduce overall production costs by employing optimization techniques. The importance of rigorous monitoring of feed quality and storage conditions cannot be overstated. Sharafi [71] emphasizes that collaboration among stakeholders in the dairy supply chain is essential for implementing best practices that ensure feed safety and quality. By integrating economic data into optimization algorithms, dairy producers can enhance their decision-making processes and improve the sustainability of their operations. As the dairy industry continues to evolve, integrating advanced optimization methods will be essential for addressing the challenges of feed formulation and promoting sustainable practices.

### 3.3. Multivariate Analysis and Data Synthesis

The use of multivariate statistical methods in feed formulation has garnered considerable attention in recent years for their capability to integrate complex data and improve the precision of feed formulations. Multivariate analysis provides a comprehensive approach to optimizing feed that directly impacts milk quality and production by combining various data types, including nutritional, environmental, and economic factors. Multivariate statistical methods, such as principal component analysis (PCA) and partial least squares regression (PLSR), are increasingly utilized to analyze the relationships between multiple variables in feed formulation. These techniques allow researchers to identify patterns and correlations within complex datasets, leading to more informed decision-making in feed formulation. For instance, O’Callaghan et al. [31] demonstrated the effectiveness of multivariate analysis in distinguishing the fatty acid profiles of milk from cows fed different supplemental feeds. Their findings indicated that specific feed choices significantly influenced milk composition, underscoring the importance of tailored feeding strategies. Similarly, Sundekilde et al. [72] utilized multivariate data analysis to explore the association between bovine milk metabolome and rennet-induced coagulation properties. Their research revealed that certain metabolites, such as choline and carnitine, could serve as indicators of milk quality, particularly in relation to coagulation properties. This highlights how multivariate analysis can provide valuable insights into the biochemical factors influencing milk quality, allowing for more precise feed formulations that enhance desirable traits.

The synthesis of data through multivariate analysis enables the identification of key factors that contribute to milk quality. For example, Botton et al. [73] employed multivariate analysis to examine the relationship between total bacterial counts, somatic cell counts, and milk composition. Their study found that higher somatic cell counts negatively correlated with lactose content, indicating that poor udder health could adversely affect milk quality. By integrating these findings into feed formulation practices, dairy producers can implement strategies that promote udder health and improve milk quality. Moreover, Macciotta et al. [74] utilized multivariate factor analysis to define new indicator variables for milk composition and coagulation properties in Brown Swiss cows. Their research demonstrated that it is possible to derive latent factors that serve as indicators of milk quality by analyzing correlated traits. This approach enhances the understanding of milk composition and facilitates the development of targeted feeding strategies that optimize milk yield and quality. The practical applications of multivariate analysis in feed formulation extend beyond academic research. For instance, Manca et al. [75] highlighted using multivariate statistics to study the correlation between milk compositional variables and mammary gland health. Their findings suggest that dairy producers can make informed decisions regarding nutritional management by monitoring these correlations, leading to improved milk quality.

The integration of multivariate analysis with emerging technologies, such as near-infrared spectroscopy (NIRS), offers promising avenues for rapid and non-invasive quality control in dairy production. Suhaini [76] demonstrated the effectiveness of Attenuated total reflectance-Fourier transform infrared (ATR-FTIR) spectroscopy combined with multivariate analysis for detecting adulterants in UHT (Ultra-high temperature processing) milk products. This approach enhances the accuracy of quality assessments and contributes to consumer safety and confidence in dairy products. The use of multivariate analysis and data synthesis in feed formulation represents a significant advancement in the dairy industry. By integrating various data types, these statistical methods enable the development of accurate and effective feed formulations that enhance milk quality and production. As the industry continues to embrace data-driven approaches, the potential for improved milk composition and consumer health benefits will only increase.

### 3.4. Decision Support Systems for Enhancing Milk Production and Feed Nutritional Composition

Considering the various optimization and analytical techniques employed to increase milk yield and enhance the nutritional composition of feed in mixed rations, decision support systems (DSS) have become indispensable tools for dairy farmers. Often implemented as software or digital tools, these systems enable farmers to interact with and utilize data-driven feed formulation techniques. DSS leverages data-driven insights to facilitate informed decision-making, optimizing key dairy management aspects, including herd health, nutrition, and overall farm sustainability. One critical feature emphasized in the literature is the preference for digital agricultural tools with user-friendly interfaces. Oyinbo et al. (2020) [77] highlight that extension agents in Nigeria favor decision support tools that are easy to use and require minimal time investment. This aligns with the broader global need for accessible agricultural technology. Such findings underscore the importance of designing DSS tools that cater to the practical needs of farmers, making adoption more likely. These systems bridge the gap between optimized yield and nutrition and the technical knowledge required, as they are designed considering farmers’ varying levels of technical expertise.

The development of mobile applications and web interfaces providing real-time information has also proven crucial for enhancing usability. Ali (2018) [78] discusses a Precision Agriculture Monitoring System with a user-friendly interface that enables farmers to monitor various farm parameters through their smartphones. This approach simplifies interaction while empowering farmers to make informed decisions based on real-time data. Advanced decision support models further exemplify the potential of DSS in enhancing dairy farm efficiency. For instance, the Farmax Dairy Pro model incorporates pasture growth potential, cow genetics, and feed management strategies to simulate and evaluate different management scenarios [79]. Similarly, the Ruminant Farm Systems (RuFaS) model offers a comprehensive framework for assessing environmental impacts and management practices, enabling farmers to make informed decisions aligned with sustainability goals [80]. These models enhance economic viability and contribute to reducing the environmental footprint of dairy operations.

**Figure 5:**
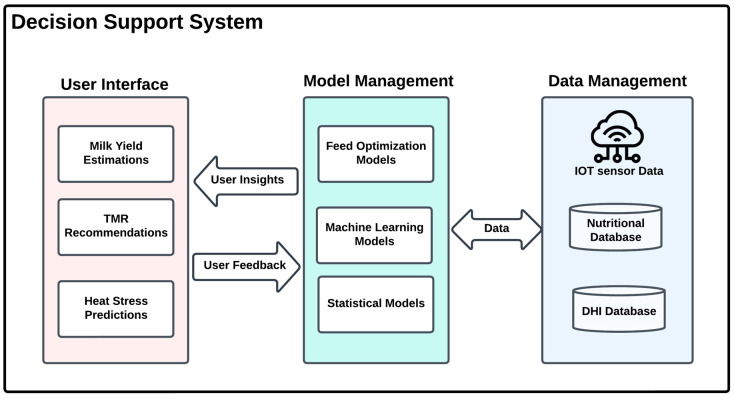
A DSS Architecture for Dairy Farmers.

As illustrated in Figure 5, the system architecture is structured into three interconnected layers, each playing a critical role in supporting data-driven decision-making in dairy farming. The Data Management Layer forms the foundation, comprising IoT Sensor Data to monitor environmental conditions in real time, a Nutritional Database offering detailed feed nutritional content, and a Dairy Herd Improvement Database consolidating historical data on herd performance, health, and breeding to guide targeted interventions. Building on this, the Model Management Layer integrates Optimization Models for cost-efficient feed strategies, Machine Learning Models to predict outcomes such as milk yield and health trends, and Statistical Models for validating predictions and providing actionable insights. The User Interface Layer delivers these insights to end-users through three outputs: Milk Yield Estimation, TMR (Total Mixed Ration) Recommendations, and Heat Stress Prediction, enabling farmers to implement effective, timely interventions [81]. The emergence of Decision Support Systems (DSS) has been pivotal in addressing the complexities of modern dairy farming. DSS continuously integrates data from multiple sources, enabling farmers to make real-time decisions based on comprehensive insights [81]. Tools such as DairyMGT provide a suite of decision-support applications that assist farmers in improving economic performance and environmental stewardship through data-driven strategies [55,82]. These systems are particularly effective in managing herd health, as they analyze data related to nutrition, disease prevalence, and reproductive performance, ultimately leading to enhanced milk yield and quality [83,84].

## 4. Challenges and Limitations in Data-Driven Feed Formulation for Milk Production

While data-driven approaches in feed formulation offer significant benefits, their full potential remains constrained by several critical challenges and limitations. These hurdles include data quality and standardization issues, technological barriers that hinder adoption, and resistance from the industry to embrace innovative practices. Addressing these challenges is essential to unlock the transformative potential of data-driven technologies in dairy production and ensure their widespread adoption and effectiveness. This section thoroughly explores these obstacles and highlights the implications for dairy farming practices.

### 4.1. Data Quality and Standardization

One of the primary challenges in data-driven feed formulation is the quality and standardization of data. The availability and reliability of data are critical for ensuring accurate feed formulations that enhance milk quality. Poor data quality can lead to inaccurate nutrient profiles, resulting in suboptimal feed formulations that negatively affect animal health and productivity. For instance, Mulyaningrum [12] highlights that inconsistent data on the nutritional value of local feed ingredients can hinder the development of effective feeding strategies, impacting livestock performance. The lack of standardized data collection methods across the industry complicates the integration of diverse datasets. Chase and Fortina [85] emphasize that variations in data quality can lead to discrepancies in feed formulation outcomes, making it difficult to compare results across different studies or farms. Establishing standardized data collection and reporting protocols is essential to improve the reliability of data-driven feed formulation practices.

### 4.2. Challenges in Measuring Critical Metrics

Data-driven approaches rely on critical metrics to feed prediction models and generate actionable insights for *Dairy* farmers. One of the significant challenges in data-driven feed formulation is accurately measuring individual feed intake, a critical metric for optimizing feed conversion efficiency and improving productivity in dairy farming. Traditional methods for measuring feed intake, such as weighing feed before and after feeding, visual estimation, and manual record-keeping, are labor-intensive, prone to inaccuracies, and unsuitable for capturing real-time, individual-level data [86,87]. Without accurate feed intake measurements, the effectiveness of data-driven approaches is limited [88]. A promising solution to this challenge lies in the use of IoT-based feed intake sensors. These sensors automate the real-time measurement of feed consumption, providing accurate and standardized data essential for informed decision-making [86,89]. By integrating seamlessly with machine learning models and optimization algorithms, feed intake sensors enable tailored feeding strategies that enhance feed efficiency and support animal health. In general, sensor technologies have made it easier to measure these metrics, but farmers are still hindered by technological barriers and resistance to change, including the relatively high investment costs associated with purchasing sensor technology [90]. Nevertheless, these sensors offer high value when integrated into decision support systems, as illustrated in Figure 5 [91].

### 4.3. Technological Barriers, Resistance to Change, and Socio-Political Dynamics

The adoption of data-driven tools in feed formulation is often hindered by technological barriers. Many dairy farms lack the computational power and infrastructure necessary to process large datasets effectively, as noted by Baldin et al. [81]. Furthermore, access to expertise in data analytics and interpretation is crucial for the effective use of data-driven systems. Many dairy producers may not have the necessary skills or knowledge to utilize these technologies fully, leading to underutilization of available data. As Neethirajan [92] highlighted, training and education are essential to equip farmers with the skills needed to effectively leverage data-driven approaches. Addressing these technological barriers is vital for promoting the widespread adoption of data-driven feed formulation practices. Resistance to adopting data-driven approaches in traditional feed formulation practices is another significant challenge. Many dairy producers are accustomed to conventional feeding strategies and may be hesitant to embrace recent technologies. This resistance can stem from a lack of understanding of the benefits of data-driven approaches or concerns about the costs associated with implementing these systems. Shivley et al. [93] discuss the importance of addressing these barriers through effective communication and demonstration of the benefits of data-driven practices. Strategies to overcome resistance may include showcasing successful case studies, providing financial incentives, and offering training programs to facilitate the transition to data-driven feed formulation. Additionally, the dairy industry must navigate the complexities of integrating recent technologies into existing practices. As noted by Knapp et al. [94], the potential for genomic technologies to enhance feed efficiency and reduce greenhouse gas emissions is substantial; however, widespread adoption will require a concerted effort to engage stakeholders and promote collaboration across the industry.

Stock and Gardezi’s [95] study provides critical insights into the challenges and limitations of data-driven feed formulation for milk production. A prominent challenge identified is the reliance on external knowledge, wherein precision agriculture (PA) shifts the locus of control from farmers’ personal, experiential knowledge to external, data-driven systems, potentially constraining their adaptability in feed formulation practices. The study also notes that trust in PA is often moralistic rather than strategic, leading to technology adoption without fully understanding long-term impacts. Concerns over data ownership, where agritech firms control farmers’ data, raise issues of privacy and decision-making autonomy. The shift to algorithmic recommendations can diminish the value of farmers’ experiential knowledge, which is crucial for effective feed formulation. These findings highlight the need for inclusive and transparent approaches to integrate data-driven feed formulation, considering the socio-political dynamics identified in the study. While data-driven feed formulation offers numerous benefits, several challenges and limitations must be addressed to maximize its potential. Ensuring data quality and standardization, overcoming technological barriers, and addressing industry resistance are critical steps toward the successful integration of data-driven approaches in dairy feed formulation.

## 5. Future Outlook for Data-Driven Feed Formulation

The future of data-driven feed formulation is poised for significant advancements, driven by emerging technologies such as artificial intelligence (AI), the Internet of Things (IoT), and big data analytics. The integration of AI and IoT technologies into feed formulation processes offers unprecedented opportunities for optimizing livestock nutrition. For grass-based dairy farms, IoT devices can monitor pasture conditions, animal behavior, and nutrient intake in real time, allowing farmers to make informed decisions about grazing management and supplementation. Shalloo et al. [96] emphasize that while implementing technology in grass-based systems presents unique challenges, such as connectivity issues, the potential benefits include improved pasture utilization and enhanced animal performance. By leveraging data analytics, farmers can adjust grazing strategies based on the nutritional quality of the grass available, ensuring that dairy cows receive a balanced diet that maximizes milk production and quality.

Navarro et al. [97] conducted a systematic review of IoT solutions for smart farming, highlighting how these technologies can facilitate real-time monitoring of animal health and feed intake. By collecting and analyzing data from various sensors, farmers can make informed decisions that enhance feed efficiency and milk quality. IoT devices can track environmental conditions, enabling adjustments in feed formulations to account for factors such as temperature and humidity, which impact animal performance [98]. Moreover, the application of big data analytics in feed formulation enables the synthesis of vast amounts of information from multiple sources, leading to more accurate and effective feeding strategies. Huang et al. [99] emphasize that the ability to analyze complex datasets can help identify optimal dietary protein levels and lipid ratios, ultimately improving feed utilization and milk production. The potential for AI to analyze historical performance data and predict future nutrient requirements further enhances the precision of feed formulations, ensuring that livestock receive the necessary nutrients for optimal health and productivity. The future of livestock nutrition is increasingly leaning towards precision and personalized feeding strategies. This approach not only improves milk quality but also enhances overall animal welfare. Kanza et al. [100] discuss how AI algorithms can analyze individual animal data to develop personalized feeding regimens that optimize nutrient intake, leading to improved milk composition and yield. Personalized nutrition has significant implications for human health as well. By enhancing the nutritional quality of milk through targeted feeding strategies, dairy producers can provide consumers with products that contain higher levels of beneficial nutrients, such as omega-3 fatty acids and vitamins. This is particularly important in addressing public health concerns related to diet and nutrition. The work of Glencross et al. [101] suggests that advancements in nutritional research can help formulate diets that not only meet the needs of livestock but also contribute to the health of consumers. As the dairy industry embraces data-driven feed formulation practices, it is essential to consider the role of policies and ethical considerations in supporting these advancements. Policymakers must create frameworks that encourage the adoption of innovative technologies while ensuring data privacy and animal welfare. For instance, the implementation of regulations that govern the use of AI and IoT in livestock management can help mitigate potential risks associated with data misuse and ensure that animal welfare standards are upheld [19].

Furthermore, ethical considerations surrounding data collection and usage must be addressed. As highlighted by Akhigbe et al. [102], the use of IoT technologies in livestock management raises questions about data ownership and privacy. Ensuring transparency in data handling and establishing guidelines for ethical data use will be crucial for fostering trust among stakeholders in the dairy industry. The future of data-driven feed formulation is bright, with emerging technologies offering significant opportunities to enhance milk quality and address human health requirements. The shift towards precision and personalized nutrition, coupled with thoughtful policy and ethical considerations, is pivotal in shaping the next generation of livestock feeding practices. To meet the increasing demands for milk production and quality, there is a pressing need for ongoing research and technological innovation in livestock nutrition. The challenges posed by climate change, evolving consumer preferences, and the necessity for sustainable practices require a robust framework for future research [103]. A comprehensive understanding of animal nutrition that incorporates environmental, economic, and social factors is essential for developing innovative solutions that enhance productivity while ensuring animal well-being and food safety [104]. Collaborative efforts across disciplines, as highlighted by the One Health approach, are crucial for addressing the multifaceted challenges in livestock production [105]. Continued investment in research and development will facilitate the discovery of novel feed ingredients and strategies that can optimize livestock health and productivity, ultimately benefiting both animals and humans alike [106].

## 6. Conclusions

This study highlights the transformative potential of data-driven approaches in enhancing milk yield and quality through precise, real-time feed formulation. By leveraging advanced techniques such as machine learning, optimization algorithms, and multivariate analysis, dairy farmers can tailor feed formulations to meet the specific nutritional needs of dairy animals, thereby improving productivity and sustainability. The integration of these technologies addresses the complexities of modern dairy animals’ nutrition, offering a promising pathway to optimize feed formulations, enhance dairy animals’ productivity, and improve the nutritional quality of products. However, challenges such as data quality, technological limitations, and industry resistance must be addressed to realize these benefits fully. Future AI, IoT, and big data analytics advancements will further refine feed formulation practices, promoting precision and personalized nutrition. Embracing these innovations, industry-wide standardization, and ethical considerations will drive the next generation of sustainable and efficient dairy farming practices.

## Data Availability

All data are embedded in the manuscript.
